# Use of the Ishii Test for screening sarcopenia in older adults: a systematic review with meta-analysis of diagnostic test accuracy (DTA) studies

**DOI:** 10.1186/s12877-024-05155-2

**Published:** 2024-07-17

**Authors:** Sabrina Gabrielle Gomes Fernandes Macêdo, Pedro Rafael de Souza Macêdo, Weslley Sales Barbosa, Álvaro Campos Cavalcanti Maciel

**Affiliations:** Postgraduate Program in Physiotherapy, Federal University of Rio Grande do Norte, Avenida Senador Salgado Filho, Natal, Lagoa Nova, Rio Grande do Norte 59078-970 Brazil

**Keywords:** Epidemiology, Geriatrics, Sarcopenia, Ishii test, Screening

## Abstract

**Background:**

The Ishii Test is recommended by the European Working Group on Sarcopenia in Older People (EWGSOP2), however the use of this technique is still little explored in the clinical context and the scientific literature.

**Objective:**

We aimed to verify the use of the Test of Ishii in screening for sarcopenia in older adults.

**Methods:**

We searched three electronic databases and two reviewers independently screened and assessed the studies. Studies with older adults (60 years or more) of both genders, no year or language restriction and which aimed to evaluate sarcopenia using the Ishii Test and another diagnostic criteria were selected. A summary of the ROC curve, sensitivity and specificity were performed using the MedCalc and SPSS software programs, respectively.

**Results:**

A total of 3,298 references were identified in the database, 278 by manually searching, and finally 11 studies were included for the review. The screening test showed good sensitivity and specificity in both genders. All studies showed values above the considered value for the Area Under the Curve (AUC) results, without discriminating power (0.500). Four studies used the original values, and five studies developed a new cut-off point. A summary of the AUC curve showed the diamond close to one, indicating that the Ishii test has good performance for screening sarcopenia (I^2^=83,66%; *p*<0.001; 95%CI: 69.38 to 91.28 for men; and I^2^=60.04%; *p*<0.001; 95%CI: 13.06 to 81.63 for women).

**Conclusion:**

The Ishii Test can be considered a useful tool for the early identification of sarcopenia in older adults. However, further studies are still needed to understand the behavior of this screening tool.

**Trial registration:**

CRD42023424392.

**Supplementary Information:**

The online version contains supplementary material available at 10.1186/s12877-024-05155-2.

## Introduction

Sarcopenia is defined as a musculoskeletal disorder characterized by reduced muscle mass and strength. It is considered an important public health problem, since its presence may lead to a progressive and generalized reduction in muscle function [1–3], and can be a precursor of physical frailty, mobility limitation, and premature death [4, 5].

Due to its great impact on the life of the affected individual (mostly older adults), the importance of an early diagnosis by identifying sarcopenia in the early stages may represent a valuable opportunity to plan and carry out interventions and slow down the progression, and consequently prevent future disabilities [6].

According to recent studies, the number of publications on sarcopenia has been growing every year [7–9], and with that different study groups have developed ways to standardize the conceptual approach to sarcopenia [1, 8, 10, 11]; however, there is a range of tools that can be used for this assessment [8].

Therefore, most of the available tools, especially those used to identify low skeletal muscle mass (SMM), which are considered the gold standard for this evaluation and strongly recommended by consensus, have high costs, radiological characteristics that restrict their use, low portability, and the need for specialized people for its operation [12–14]; all of which makes it practically impracticable to apply the standard algorithms, and consequently makes it difficult to identify sarcopenia early in community-dwelling, institutionalized or hospitalized older adults [12–14].

By analyzing the need to explore simple, easy-to-operate, and low-cost alternatives, some studies developed valid screening tests and waited to identify sarcopenia as early as possible [15]. An alternative that can be used to quickly identify sarcopenia is the Ishii Test. It was developed by Ishii Ishii et al. (2014), and uses an equation-derived score based on three items: age, handgrip strength, and calf circumference, which are easy to perform to calculate the probability of developing sarcopenia [15, 16]. According to Ishii et al. [16] and Zhu et al. [12], the score generated by the test can be used to predict future adverse events, in addition to presenting high sensitivity and specificity for identifying sarcopenia. The final score is also associated with worsening in the general functional status of older adults [17], which makes it strongly predictive of the onset of sarcopenia in older adults from different contexts [13, 18].

Although the Ishii Test is recommended by the European Working Group on Sarcopenia in Older People (EWGSOP2) [19], the use of this technique is still little explored in the clinical context and the scientific literature. In a systematic review carried out in 2023 by Huang et al. [20], it was found that the Ishii Test had good sensitivity and specificity for screening sarcopenia when compared to the other consensus.

According to Tang et al. [15], this can be explained because the test was developed in the Japanese population and only validated in the Chinese population until mid-2018; moreover, few changes have been made regarding the validity and use in other regions of the world. Therefore, despite being recommended by the consensus most used by researchers and clinicians, it is still necessary to investigate the use of the test and its final evaluation in different populations.

Given the low number of studies on the subject, the question about its validity in older adults around the world, and the absence of reviews which show the importance of this evaluation method, the present systematic review has the main objective to conduct a literature survey on the use of the Ishii test for screening sarcopenia in older adults in different contexts. Associated with this, this work will verify the accuracy of the Ishii Test according to the reference measure to which it was compared.

## Methods

The protocol for this review was registered in the Prospective Register of Systematic Reviews (PROSPERO) (CRD42023424392). We followed the recommendations of Preferred Reporting Items for Systematic Review and Meta-Analyses of Diagnostic Test Accuracy Studies guidelines (PRISMA-DTA), and the Joanna Briggs Institute’s (JBI) Assessing the Methodological Quality of Systematic Reviews (AMSTAR) [21–24]. The research question which conducted this review was based on the PIRD (population, index test, reference test, and diagnosis of interest) acronym, namely: *“Can the Ishii test be used to screen for sarcopenia, compared to other diagnostic methods, in older people inserted in different contexts?”*

### Eligibility criteria

Eligibility criteria were established according to the PICO strategy: I) Studies with older adults (60 years or more) of both genders. The choice of 60 years as the minimum age limit can be explained because the World Health Organization establishes this parameter to consider the older population in developing countries [25]; II) no year or language restriction; III) Studies which aimed to evaluate sarcopenia using the Ishii Test and another diagnostic criteria; VI) Studies which examined the diagnostic accuracy of the Ishii test against a widely accepted diagnostic criteria (i.e. sarcopenia consensus).

Articles were excluded if they: a) assessed sarcopenia using other diagnostic methods; b) were carried out with participants younger than 60 years old; c) were not published in a peer-reviewed journal and published as editorials, letters, comments on previously published articles, review articles with data based on meetings or repositories of dissertations and theses, and all gray literature (i.e. congress proceedings) were also excluded.

### Search strategy and research information

The search was conducted in three databases: MedLine, Web of Science, and Scopus, from inception until August 2023. We included terms with wider meanings in addition to “Ishii” to avoid search omissions [26]. The search strategy was developed using the Medical Subject Heading (MeSH) descriptors considering the following standardized formula for all databases: Ishii AND sarcopenia AND screening AND accuracy AND (older or "older people" OR elderly).

### Selection process and data extraction

The results after searching the databases were exported to Rayyan^®^ (Qatar Computing Research Institute – Data Analytics, Doha, Qatar), a web-based software which facilitates collaboration between reviewers during the study selection process. Titles, abstracts, and full text were assessed by two investigators independently to identify eligible studies (WSB and PRSM). Any disagreements were resolved by a third researcher, who evaluated the study and made the final decision (SGGFM). After inclusion of articles, the list of references was manually checked by reviewers in search of new studies that met the eligibility criteria.

Data from the included articles were extracted by a reviewer (PRSM) and checked by a second examiner (SGGFM). At this stage, a standardized form was used in Excel^®^ which contained information presented in Tables 1 and 2 (supplementary file 01).

**Table 1:** Study characteristics

**Author, year, and country**	**Design**	**Place of study**	**Population (N)**	**Mean age**	**Ishii formula**	**Cut-off point for Ishii Test**	**Prevalence or score mean by Ishii Test**	**Sarcopenia prevalence of the screening method used for comparison (% or N)**
Alsadany et al. (2021) [27], Egypt	Cross-sectional	Hospital	127 older adults (65 men and 62 women)	Sarcopenic men: 70.23 ± 6.37 Non-sarcopenic men: 62.03 ± 3.01 Sarcopenic women: 67.33 ± 6.7 Non-sarcopenic women: 63.5 ± 3.1	Yes^a^	Original and new cut-off point: ≤115 for females and ≤100 for males.	NR	EWGSOP2: Men: N: 31 (24.4%) Women: 15 (11.8%)
Chen et al. (2021) [18], China	Cross-sectional	Community	941 older adults (462 men and 479 women)	NR	Yes^a^	Original and new cut-off point (Youden index): 102 for females and 95 for males.	NR	AWGS 2019 Criteria: Total: 18.38% Men: 19.91% Women: 16.91%
Ding et al., (2023) [28], China	Cross-sectional	Hospital	215 older adults (both genders)	Total: 60.5	Yes^a^	New cut-off point 102.3 for males and 98.3 for females calculated by the Youden index	NR	AWGS 2019 Sarcopenia: 47.9% Severe sarcopenia: 18.6%
Erdogan et al., (2021) [9], Turkey	Cross-sectional	Geriatric ambulatory	1,701 older adults	Men: 75.9 ± 6.7 Women: 74.1 ± 7.1	Yes^a^	Original	4.20%	EWGSOP2 Probable sarcopenia Men: 18.3% Women: 8.9% Confirmed sarcopenia: Men: 1.8% Women: 0.2% Severe sarcopenia: Men: 0.6% Women: 0.2%
Huang et al., (2023) [29], China	Cross-sectional	Community	966 older adults (both sexes)	Non-sarcopenic: 71.00 Sarcopenic: 78.00	Yes^a^	Original	NR	AWGS (2019) – sarcopenia (*n*=81) and non-sarcopenia (*n*=597)
Ishii et al. (2014) [16], Japan	Cross-sectional	Community	1,971 older adults (977 men and 944 women)	Non-sarcopenic: 72.2 ± 5.0 Sarcopenic: 78.4 ± 5.5	Yes^a^	Original	Sarcopenic: Men: 9.6% Women: 12.7%	EWGSOP Men: 14.2% Women: 22.1%
Li et al., (2019) [14], China	Cohort prospective	Hospital	138 older adults	Non-sarcopenic: 68.9±8.5 Sarcopenic: 74.5±9.7	Yes^a^	Original	*N*=36	AWGS: *N*=35
Li Min et al., (2018) [30] , China	Cross-sectional	Community	122 older adults (both sexes)	71.8±7.8	Yes^a^	Original	NR	AWGS – 30.33%
Lin et al. (2021) [13], China	Cross-sectional	Nursing home care	199 older adults (97 men and 102 women)	NR	NR	Original	NR	AWGS 2019 Criteria: Total 48.7%
Locquet et al., (2017) [31], Belgium	Cross-sectional	Community (from SarcoPhAge)	306 older adults (both sexes)	Men: 75.0±5.9 Women: 74.7±5.9	Yes^a^	New cut-off point developed using the Youden index and compared by: EWGSOP: 112.3 IWGS: > 111.1 Society of Sarcopenia, Cachexia and Wasting Disorders: 117.8 AWGS: 117.8 FNIH: 128.5	NR	*Men:* EWGSOP criteria: *N*= 19 IWGS criteria: *N*=16 Society of Sarcopenia, Cachexia and Wasting Disorders criteria: *N*= 8 AWGS criteria: *N*= 5 FNIH criteria: *N*=7 *Women:* EWGSOP criteria: *N*=32 IWGS criteria: *N*=21 Society of Sarcopenia, Cachexia and Wasting Disorders criteria: *N*= 10 AWGS criteria: *N*= 12 FNIH criteria: *N*= 15
Zhu et al., (2022) [12], China	Cross-sectional	Nursing home care	199 older adults (both sexes)	Non-severe sarcopenic: 71.17 (±8.54) Severe sarcopenic: 79.07 (±9.04)	NR	New cut-off point 130 for both genders calculated by Youden index	NR	AWGS (2019) – Severe sarcopenia: 33.7%.

*NR* Not reported, *EWGSOP* European Working Group on Sarcopenia in Older People, *IWGS* International Working Group on Sarcopenia, *AWGS* Asian Working Group for Sarcopenia, *FNIH* Foundation for the National Institutes of Health

^a^Ishii formula: score in men, 0.62 × (age – 64) – 3.09 × (grip strength – 50) – 4.64 × (calf circumference – 42); score in women, 0.80 × (age – 64) – 5.09 × (grip strength – 34) – 3.28 × (calf circumference – 42)

**Table 2:** The accuracy of the Ishii score chart in predicting sarcopenia

**Study**	**Accuracy values**				
	**Sensitivity**	**Specificity**	**PPV**	**NPV**	**AUC (95% IC)**
	**%**				
Alsadany et al. (2021) [27]	Men				
	87	75	75	86	0.93 (0.83 to 0.98)
	Women				
	80	78	93	76.6	0.86 (0.79 to 0.98)
Chen et al., (2021) [18]	Men				
	Original cut-off point				
	64.94	85.46	64.94	92	NR
	New cut-off point				
	70.65	81.35	70.65	92	0.81 (0.75 to 0.86)
	Women				
	Original cut-off point				
	46.91	93.22	46.91	90	NR
	New cut-off point				
	75.31	79.9	75.31	94	0.84 (0.80 to 0.89)
Ding et al., (2023) [28]	Men				
	93.2	59.1	79.07	83.8	0.83 (0.75 to 0.90)
	Women				
	93.3	64.7	52.83	95.6	0.84 (0.77 to 0.93)
Erdogan et al., (2021) [32]	Probable sarcopenia				
	84 (78.1–88.9)	86.1 (84.2–87.9)	84 (78.1–88.9)	97.6	84 (78.1 to 88.9)
	Confirmed sarcopenia				
	100 (71.5–100)	83.9 (82–85.7)	100 (71.5–100)	100	100 (71.5 to 100)
	Severe sarcopenia				
	100 (47.8–100)	84.6 (82.6–86.3)	100 (47.8–100)	100	100 (47.8 to 100)
Huang et al., 2023 [20, 29]	Total				
	71 .0	75.0	28.0	95.0	0.73 (0.67 to0.79)
Ishii et al. (2014) [16]	Men				
	84.9	88.2	84.9	97.2	0.940 (0.92-0.95)
	Women				
	75.5	92.0	75.5	93.0	0.91 (0.88 to0.93)
Li et al., (2019) [14] AWGS	Men				
	88.9	70.6	NR	NR	0.87 (0.61 to 0.83)
	Women				
	77.8	68.6	NR	NR	0.78 (0.65 to 0.91)
Li Min et al., (2018) [30] AWGS	Men				
	88.0	73.0	NR	NR	0.91 (0.82-1.00)
	Women				
	82	82	NR	NR	0.85 (0.76-0.95)
Lin et al. (2021) [13]	Men				
	94.83	56.41	94.83	0.88	0.86 (, 0.78-0.94)
	Women				
	82.05	85.71	82.05	0.89	0.85 (0.77-0.94)
Locquet et al. (2017) [31]	EWGSOP criteria				
	89.70	80.9 (76.5–85.3)	89.70	96.3 (94.2–98.4)	0.85 (0.80–0.90)
	EWGSOP2 criteria				
	84.3 (80.2–88.4)	77.7 (73.0–82.4)	84.3 (80.2–88.4)	97.7 (96.0–99.4)	84.3 (80.2–88.4)
	IWGS criteria				
	86.8 (83.0–90.6)	74.3 (69.4–79.2)	86.8 (83.0–90.6)	97.7 (96.0–99.4)	86.8 (83.0–90.6)
	Society of Sarcopenia, Cachexia, and Wasting Disorders criteria				
	100.0 (100–100)	74.1 (69.2–79.0)	100.0 (100–100)	100.0 (100–100)	100.0 (100–100)
	AWGS criteria				
	100.0 (100–100)	74.9 (70.0–79.8)	100.0 (100–100)	99.1 (98.0–100)	100.0 (100–100)
Zhu et al. (2022) [12]	Total				
	89.6	83.3	89.6	0.94	0.89 (0.84-0.93)
	Men				
	85	77.2	85	0.88	0.82 (0.74-0.91)
	Women				
	96.3	88	96.3	0.92	0.83 (0.73-0.92)

*NR*: Not reported, *EWGSOP* European Working Group on Sarcopenia in Older People, *IWGS* International Working Group on Sarcopenia, *AWGS* Asian Working Group for Sarcopenia, *FNIH* Foundation for the National Institutes of Health, *PPV* Positive predictive value, *NPV* Negative predictive value, *AUC* Area under the curve

### Methodological quality

The Quality Assessment of Diagnostic Accuracy Studies (QUADAS-2) was used to assess the risk of bias in four dimensions (patient selection, index text, reference standard and low and timing). Based on the responses obtained, the risk of bias can be classified as low, high, or unclear. Two independent reviewers (WSB and PRSM) assessed the quality of the included studies.

### Data analysis

Descriptive data are presented in tables and graphs. A summary receiver operating characteristic (sROC) curve was constructed with the included studies that presented AUC data and confidence interval (95%), according to gender and considering all reference consensuses. Only studies which presented AUC and 95%CI values for both sexes were considered to carry out this test, with 9 studies being considered at this stage.

According to Spick et al. [33], ROC curves show the trade-off between sensitivity and specificity, whereby a test can be more sensitive (by over-diagnosing disease) at the cost of being less specific (more false positives), and vice versa. A test that was 100% sensitive and 100% specific would generate an area under the curve (AUROC) of exactly 1, and generally values closer to 1 indicate better diagnostic performance.

Finally, the degree of heterogeneity of the studies included in the sROC was measured using I^2^, with I^2^ values of 25, 50, and 75%, indicating low, moderate, and high heterogeneity, respectively [34]. A Z-test was performed to compare the pooled sensitivity or specificity of Ishii Test for each gender, according to diagnostic criteria (p ≤ 0.05 indicated significant differences). All analyzes were performed using the MedCalc 22.09^®^ program.

## Results

### Selection of studies

**Fig. 1:**
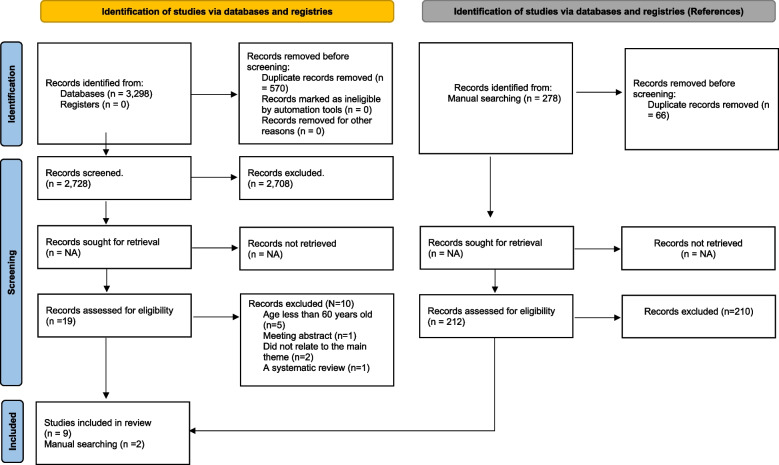
Flowchart of studies found for this review

A total of 3,298 references were identified in the initial search carried out in the databases, of which 570 were excluded due to duplicity. After reading the titles and abstracts, 2,708 articles were excluded and a total of 19 had their full text read to check eligibility. At this stage, 10 references were excluded for not meeting the criteria adopted for this review. A manual search was then carried out and two articles included in this work were checked. Details on the study identification procedure can be seen in Fig. 1 and the main characteristics of the included studies are summarized in Table 01.

### Characteristics of selected studies

A total of 11 studies were selected. A total sample of 6,885 participants of both sexes were assessed, with a mean age of at least 63 to 84 years old. Studies were carried out with older adult residents in different places, three of which were carried out in hospitals [14, 15, 27], five in communities [16, 18, 31, 35], two in nursing home care [12, 13] and one in ambulatory [9]. The characteristics of the study can be found in Table 1.

### Ishii formula and cut-off points used

The original study [16], which is also included in this systematic review, developed a mathematical equation comprising the variables of age, handgrip strength and calf circumference according to gender, with cut-off points of ≥ 105 points for men and ≥ 120 points for women.

Studies by Erdogan et al. [9], Lin et al. [13], and Huang et al. [20] only used the values determined by the original study (≥105 points for men and ≥ 120 points for females). Thus, [12, 18, 27, 31] developed new cut-off points based on their populations using the Youden Index statistical method. Li Min et al. [14] developed new cut-off points; however, the values found were the same as the original.

Therefore, we see that the new values found vary, being lower than the original for men and women in the studies by Alsadany et al. [27], Chen et al. [18], and Ding et al. [28], and higher for both sexes in the study by Zhu et al. [12]. Locquet et al. [31] developed new values using the different sarcopenia consensuses as a standard for comparison [1, 10, 11, 36, 37], which used values ranging from 11.1 to 128.5 for both men and women.

### Number of cases and prevalence of sarcopenia

The number of cases and the prevalence of older adults with sarcopenia using the Ishii Test were only observed in the studies by Erdogan et al. (2021), Ishii et al. (2014), and Li Min et al. (2019), and compare the values found with the international consensus [1, 10, 11, 36, 37]. It was seen that when using the Ishii Test, the prevalence of sarcopenia was lower when compared to the gold standard method, except in the study by Li Min et al. (2019), who verified the difference of only one case compared to the AWGS (*N*=36 vs *N*=35, respectively). In observing the prevalence of sarcopenia using the consensus as diagnostic criteria (L.-K. Chen et al., 2014; Cruz-Jentoft et al., 2010; Fielding et al., 2011; Morley et al., 2011; Studenski et al., 2014)(L.-K. Chen et al., 2014; Cruz-Jentoft et al., 2010; Fielding et al., 2011; Morley et al., 2011; Studenski et al., 2014), it is noted that there is a variation in the results, depending on the method used.

### Comparative results of sensibility and specificity of the Ishii Test based on different diagnostic criteria

Figures S1 A and B and Figure S2 C and D (supplementary material) show the summary comparison of sensitivity and specificity results between the test measure (Ishii’s Formula) and the sarcopenia consensus according to gender. It can be observed that the Ishii Test showed greater sensitivity when compared to AWGS1 and greater specificity when compared to EWGSOP1, for both men and women. There was no significant difference between the variables for the Z test results.

### Screening probability using the Ishii test

In analyzing the results of the diagnostic accuracy of the Ishii Test (sensitivity, specificity, positive predictive value (PPV), negative predictive value (NPV), and Area Under the Curve (AUC)) for screening sarcopenia in the articles included (Supplementary file 01).

Considering the original cut-off values according to gender, the screening test was more sensitive for men and more specific for women in the studies by Ishii et al. (2014) and Lin et al. (2021), whereas in the study by Zhu et al. (2022) there was a greater sensitivity and specificity for women; while in Li Min et al. (2019) there was better performance of accuracy for men. However, despite differences in results between genders, all studies show that the Ishii Test has good sensitivity and specificity in men and women, proving capable of identifying the presence of sarcopenia when necessary.

In the study by Huang et al. (2023), the sensitivity and specificity values were lower than 75% when compared to other screening methods; however, the test proved to be effective in screening for sarcopenia in cancer patients. When looking at the PPV values for this study (26%), this means that the probability of presenting the disease when the test is positive is lower.

Locquet et al. [31] showed accuracy results according to different criteria (EWGSOP1, EWGSOP2, IWGS, Society of Sarcopenia, Cachexia and Wasting Disorders and AWGS) [1, 10, 11, 36, 37], and it is possible to observe that the Ishii Test had 100% sensitivity when compared to the Society of Sarcopenia, Cachexia and Wasting Disorders criteria and the AWGS and presented better specificity (80.9%) with the EWGSOP2.

All values regarding NPV are greater than 87%, which indicates a good probability that the individual is not sarcopenic in case the test is negative. However, PPV values are low, indicating that the probability of having sarcopenia when the test is positive is lower; in addition, there is a variation in these values (14.5 – 46.7) depending on the criterion used (consensus).

For new cut-off points, five studies [14, 18, 27, 28, 32] developed new values for sarcopenia screening. In Chen’s study, it is possible to analyze the comparison between the accuracy according to the new and original values, and it is observed that sensitivity for both genders is less than 70% when using the values established by Ishii et al. [16], which changes when considering the new cut-off values; however, the sensitivity reduces when comparing with the original.

The study by Alsadany et al. [27] uses the values of 115 and 110 and observes greater sensitivity in men and greater specificity in women. Erdogan et al. [32] verified the diagnostic capacity of the Ishii test in the different degrees of sarcopenia and observed that there was 100% sensitivity and NPV for confirmed and severe sarcopenia, however, the PPV value was lower than the other studies analyzed. The new cut-off values in Ding’s study present good sensitivity for both sexes; however, the specificity of the test was low when compared to other studies analyzed, especially for males. In the study by Li-Min et al. (2019), the cut-off values were equal to those found by Ishii et al. (2014), and showed good specificity and sensitivity in screening for sarcopenia.

## Summary of ROC curve

All studies showed AUC values above the considered “value without discriminating power (0.500)”; therefore, it is possible to say that the Ishii Test has good performance for identifying sarcopenia in the evaluated older people.

**Fig. 2:**
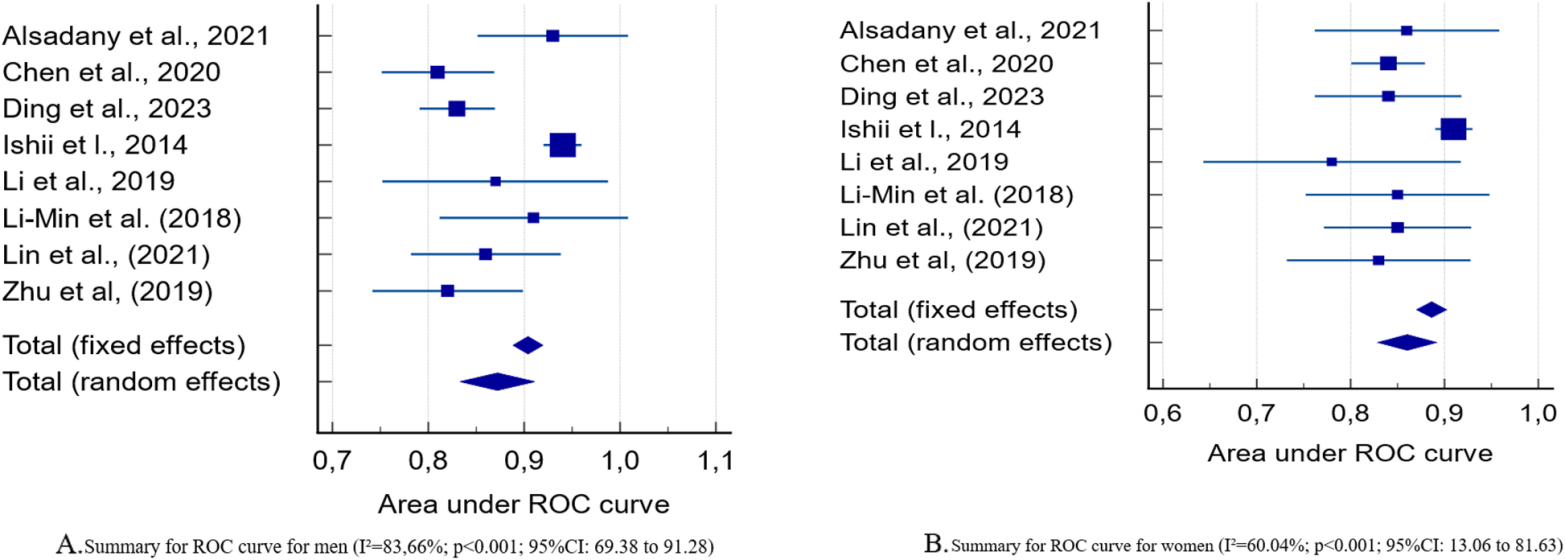
**A** Summary of the ROC curves for men; **B** Summary of the ROC curves for women

Figure 2A and B represent a summary of the ROC curves of the studies included according to gender. For this analysis, only articles that presented AUC Curve results for men and women were considered. It is possible to observe in both figures that the diamond is close to the value 1, indicating that the Ishii test has good performance for screening sarcopenia.

Regarding the heterogeneity of the studies (I^2^ index), an I^2^ value equal to 60% was found for both women and 80% for men, which shows moderate and high heterogeneity, respectively.

### Methodological quality

Table 3 summarizes identified risks of bias for the studies reviewed in this work established using the QUADAS-2 framework, with the proportion of studies by each risk category shown in Fig. 1. Of these studies, the index test and reference standard were low risk of bias in almost studies, whereas patient selection and flow and timing the risk of bias were high.

**Table 3:** Risks relating to bias (QUADAS-2)

**Study**	**RISK OF BIAS**			
	**PATIENT SELECTION**	**INDEX TEST**	**REFERENCE STANDARD**	**FLOW AND TIMING**
Alsadany et al., 2021 [27]				
Chen et al., 2021) [18]				
Erdogan et al., 2022 [9]				
Ding et al., 2023 [28]				
Ishii et al., 2014 [16]				
Huang et al., 2023 [20, 29]				
Li et al., 2019 [14]				
Lin et al., 2021 [13]				
Locquet et al., 2017 [31]				
Li Min et al., 2018 [30]				
Zhu et al., 2022 [12]				

Low Risk  High Risk

## Discussion

This systematic review had the main objective to verify the use of the Ishii Test in older adults living in different contexts, whether community or not; in addition, the validity of this screening tool and the prevalence of sarcopenia when used in this population group were verified.

### Main findings

We observed that most of the studies were developed in China, except for the studies by Erdogan et al. [32], Alsadany et al. [27] and Locquet et al. [31]; therefore, the validity of the test is practically restricted to the Asian population. In turn, this review reinforces the need to carry out more studies using this diagnostic method. Corroborating this point of view, a review by Dent et al. [38] concludes that despite the Ishii Test being an alternative, further validation studies are still needed, mainly outside the Asian region. Furthermore, this work reinforces the need to encourage the use of this easily applicable and low-cost measure by health professionals, allowing rapid screening for sarcopenia and avoiding future complications in the older adult population.

### Prevalence and number of cases

Comparing the prevalence and number of cases of sarcopenia using the gold standard methods and the Ishii Test, it is observed that the prevalence is lower for the latter. This can be explained because according to some studies by Alsadany et al. [27] and Chen et al. [18], the Ishii Test works better to rule out those cases at risk of sarcopenia, which is different from what is proposed by consensus and gold standard methods that aim to identify those who are really affected by the disease (rule-in); therefore, the chance of finding truly sarcopenic people using this screening test is lower when compared to traditional methods.

There is also heterogeneity of values in analyzing the prevalence of sarcopenia when using different consensuses. This corroborates with what was seen by Fernandes et al. [8] when analyzing studies carried out in communities of older adults from different locations, and observed a variation from 2.4 - 35.05%. This prevalence range was also observed in older residents in nursing home care and institutionalized [17, 39, 40].

According to previous studies [41–44], this variation in values depends on the cut-off points and consensus used. As an example, we can highlight the study by Qian et al. (2024), which observed a difference in prevalence in older Chinese adults when using the AWGS 2019 (70.5%) and the AWGS 2014 (11.22%), and which can be explained due to the increased cut-off values for gait speed and handgrip strength in men. Another study carried out by Fernandes et al. [8] observed the same heterogeneity pattern in the prevalence of sarcopenia depending on the diagnostic methods used. Therefore, we can reinforce that different evaluation methods and instruments and the studied population influence the cases of sarcopenia.

### Ishii test, mathematical formula, and cut-off point

The included studies used the Ishii Equation, which is based on the following variables: age, calf circumference, and handgrip strength. According to Ishii et al. [16], the choice of these variables was due to the significant correlations about strength and muscle mass, essential elements for the screening of sarcopenia, and which are also used in the gold-standard diagnostic criteria.

However, some studies have developed new cut-off values based on their specific populations, so according to Arango-Lopera et al. [45], it is possible to produce inconsistent results when using original cut-off values in genotype and phenotypically distinct populations; associated with this, the calf circumference and handgrip strength variables are sensitive to ethnic differences, such as gender, race and body composition, which may influence the identification of sarcopenia [46–48].

It is clear in the literature that the cut-off values influence the ability to identify or not the studied outcome, increasing or reducing the sensitivity and specificity of the test. Corroborating this information, Chen et al. [18] shows that when using the new cut-off values, the test’s prediction sensitivity increased from 46.9 to 75.3% in women and from 64.9 to 70.65% in men. Similar to these findings, Shuyue Lou et al. [49] also found that the NPV, which is directly linked to test sensitivity, was 98% in both sexes when using new cut-off values.

In studies by Edorgan et al. [32] and Locquet et al. [31], sensitivity and NPV reached 100% in certain situations when developing new reference values, which may mean that the new values created were too rigorous. Moreover, there is a risk of specificity and PPV having lower values with sensitivity values so high, increasing the risk of false positives [50].

### Diagnostic accuracy

#### Sensitivity and specificity

The Ishii Test showed high sensitivity and specificity in almost all evaluated studies. Like our findings, the review carried out by Huang et al. (2023) shows that the Ishii score has superior specificity (0.85, 95%CI; 0.77–0.90) for diagnosing sarcopenia. Only Ding et al. [28] presented low specificity values for both men and women, suggesting that there was a greater possibility of overestimation of sarcopenia in the studied population, which, unlike other studies, was composed of cancer patients.

According to McNamara et al. [51], these qualities make the test a good option for screening; therefore, greater sensitivity shows that the Ishii Test can identify individuals at risk of developing sarcopenia, enabling screening and early interventions, being beneficial for clinical practice and for the health of the older adult population [31, 32].

Good sensitivity and specificity results are expected when using the Ishii Test when compared to other screening instruments such as the SARC-F [52] or the Screening grid [53], since the variables contained in the equation are present or indirectly linked to what is used in the original assessment. Based on these observations, it is possible to affirm that the Ishii Test is valid and reliable in screening sarcopenia in the older adults evaluated associated with poor functional status [17, 54].

Overall, the Ishii test exhibited high sensitivity and accuracy, which may be attributable to the fact that it takes handgrip strength, which is itself a diagnostic criterion for sarcopenia, into consideration [55]. While handgrip strength is just one component of the overall diagnosis of sarcopenia, it can be assessed in an inexpensive, convenient, and portable manner in contrast to DXA and BIA, highlighting its potential value as a screening tool [20, 55].

#### PPV, NPV, and AUC

All analyzed studies showed satisfactory NPV values (accompanied by good sensitivity), which allows us to say that the Ishii Test presents good performance in identifying those who do not suffer from sarcopenia (rule-out). For Trevithan (2017), a high NPV value is desirable, which implies that false negative cases are minimized. The studies by Ding, Ishii, Erdogan, Zhu, Lin, and Li Min [12–14, 16, 28, 32] showed high PPV values, indicating that the Test was able to identify those who presented the condition in these specific populations.

However, the PPV values in Erdogan et al. [32], Locquet et al. [31], Huang et al. [29], and Chen et al. [18] were low, which strengthens the hypothesis that the Ishii Test has a better performance when “excluding” (rule-out) the possible cases. Therefore, according to Erdogan et al. [32] and Chen et al. [18], even with low rates of PPV, the tool would not ignore cases of sarcopenia; however, individuals could be detected as sarcopenic when they do not have the disease, leading to the appearance of false positives. Nevertheless, in some cases, especially in screenings and that no damage is harmful, the appearance of mistakenly positive cases may be acceptable, thus protecting the older adult population from the target condition [56].

Locquet et al. [31] showed that the PPV has varied values depending on the reference method used, being higher when compared to the EWGSOP. A possible explanation for this is that the Ishii Test equation was developed based on the EWGSOP. In addition, it is worth mentioning that each screening algorithm considers other diagnostic criteria [8], such as gait speed, which may influence the identification of sarcopenia and agreement with the screening test. Huang et al. [29] presented the lowest PPV value among the studies; however, unlike the results of this review, this study compares the Ishii Test with a new assessment tool which is still in the process of being developed. Validation (AB3C model) and possibly the nature of this tool, which contains subjective measures such as the assessment of dietary diversity and the definition of exercise intensity, may have led to inferior results.

Finally, the AUC was higher in all of the studies included than what is considered acceptable [57], presenting values between 0.8-0.9, thus indicating that the Ishii Test has an excellent discriminating property between sarcopenic and non-sarcopenic patients in both sexes. This information is confirmed when observing the summary of the ROC curve presented in Fig. 2A and B, where values close to 1 show good performance.

**Fig. 3:**
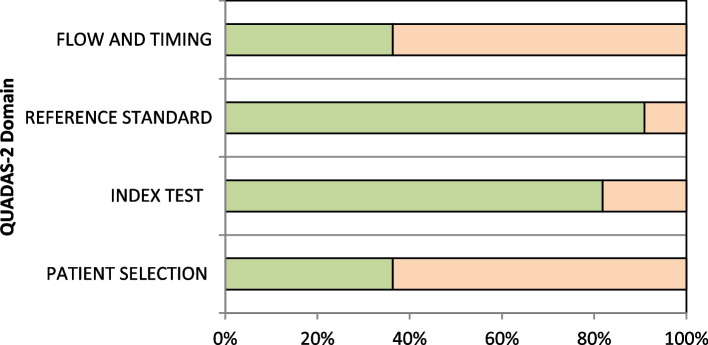
Proportion of studies with low, high, or unclear risks of bias

The moderate and high heterogeneity present in our results can be explained due to the nature of the population included in the primary articles, since there are studies carried out in different regions of the world with older people living in communities and hospitalized. As a result, they present different sociodemographic and body characteristics which will affect this result. A similar observation was made by Lu et al. [26] when verifying the accuracy of the SARC-F, another sarcopenia screening instrument, in older adults from different contexts Fig. 3.

In summary, it was possible to analyze the use of the Ishii Test in this review and identify the sensitivity, specificity, predictive values, as well as its applicability in different contexts. Furthermore, despite the variation in the values found, it can be observed that this screening method is considered a valid option for identifying sarcopenia in the older adult population. Different guidelines recommend different screening tools. If the accuracy of different screening tools can be analyzed and compared under the same diagnostic criteria, it may provide new ideas for researchers to perform sarcopenia screening in different areas, thus promoting sarcopenia screening in older adults [25].

### Limitations and strengths

This review has important restrictions. First, the lack of studies evaluating the Ishii Test in other regions makes it difficult to extrapolate its use to older people living in America and Europe; therefore, there is a need for external validation studies. Second, the small number of participants included in the selected articles and the eligibility criteria may influence the results found and make it difficult to extrapolate the use of the screening tool to other groups. Third, the use of variables sensitive to ethnic differences and the use of handgrip strength, where the use of a dynamometer is necessary for its evaluation, can reduce the ability to use the Ishii Test.

Finally, the absence of information such as true positives (TP), false positives (FP), true negatives (TN), and false negatives (FN) made it difficult to carry out a meta-analysis about sensitivity and specificity. Most of the studies analyzed presented negative and positive predictive values; however, according to Trikalinos et al. [58], predictive values depend on extremely broad prevalence estimates, so it is rarely meaningful to combine them in a meta-analysis.

As strengths, we saw that this is the first systematic review which analyzes the use of the Ishii Test in the older adult population. In addition, it was possible to carry out a detailed survey of the cut-off values and the diagnostic accuracy of the test according to the place of residence of the older person, showing in which situations the Ishii Test presents a better performance for detecting sarcopenia.

## Conclusion

The Ishii Test can be considered a useful tool for the early identification of sarcopenia in older adults; however, further studies are still needed to understand the behavior of this screening tool. In addition, little is known about the use of the Ishii Test in older adults living in long-term care or nursing home care, so it is worth emphasizing the need for further investigation into these same experiences.

Despite some limitations regarding its use, the Ishii Test can have a positive impact on clinical practice, since it is a low-cost test with easy applicability. Therefore, the use of the Ishii Test will enable early identification of sarcopenia, allowing quick and direct action, facilitating management of the older adult by the health professional, and helping to develop and strengthen public policies to minimize the deleterious effects caused by sarcopenia.

### Supplementary Information

Supplementary Material 1.

Supplementary Material 2. 

Supplementary Material 3. 

## Data Availability

No datasets were generated or analysed during the current study.

## Abbreviations

AUC: Area Under the Curve
EWGSOP2: European Working Group on Sarcopenia in Older People
FN: False negatives
FP: False positives
JBI: Joanna Briggs Institute
NPV: Negative predictive value
PPV: Positive predictive value
PRISMA-DTA: Preferred Reporting Items for Systematic Reviews and Meta-Analyses-Diagnostic Test Accuracy
PROSPERO: Prospective Register of Systematic Reviews
TN: True negatives
TP: True positives
