# Supplementary material for: Use of the Ishii Test for screening sarcopenia in older adults: a systematic review with meta-analysis of diagnostic test accuracy (DTA) studies
Source: BMC Geriatr. 2024 Jul 17;24:609. doi: 10.1186/s12877-024-05155-2 (PMC11253494; doi:10.1186/s12877-024-05155-2)
Supplement: Supplementary file 1 — Supplementary Material 1. [file 12877_2024_5155_MOESM1_ESM.docx]

| **Study** | **Accuracy values** | | | | |
| --- | --- | --- | --- | --- | --- |
|  | **Sensitivity** | **Specificity** | **PPV** | **NPV** | **AUC (95% CI)** |
|  | **COMPARE TO EWGSOP1** | | | | |
| Ishii et al. (2014) | Men | | | | |
|  | 84.9 | 88.2 | 84.9 | 97.2 | 0.940 (0.92-0.95) |
|  | Women | | | | |
|  | 75.5 | 92.0 | 75.5 | 93.0 | 0.91 (0.88 to0.93) |
|  |  |  | **COMPARE TO EWGSOP2** |  |  |
| Alsadany et al. (2021) | Men | | | | |
|  | 87 | 75 | 75 | 86 | 0.93 (0.83 to 0.98) |
|  | Women | | | | |
|  | 80 | 78 | 93 | 76.6 | 0.86 (0.79 to 0.98) |
| Erdogan et al. (2021) | Probable sarcopenia | | | | |
|  | 84 (78.1–88.9) | 86.1 (84.2–87.9) | 84 | 97.6 | 84 (78.1 to 88.9) |
|  | Confirmed sarcopenia | | | | |
|  | 100 (71.5–100) | 83.9 (82–85.7) | 100 | 100 | 100 (71.5 to 100) |
|  | Severe sarcopenia | | | | |
|  | 100 (47.8–100) | 84.6 (82.6–86.3) | 100 | 100 | 100 (47.8 to 100) |
|  | **COMPARE TO AWGS1** | | | | |
| Huang et al. (2023) | Total | | | | |
|  | 71 .0 | 75.0 | 28.0 | 95.0 | 0.73 (0.67 to0.79) |
| Li et al. (2019) | Men | | | | |
|  | 88.9 | 70.6 | NR | NR | 0.87 (0.61 to 0.83) |
|  | Women | | | | |
|  | 77.8 | 68.6 | NR | NR | 0.78 (0.65 to 0.91) |
| Li Min et al. (2018) | Men | | | | |
|  | 88.0 | 73.0 | NR | NR | 0.91 (0.82-1.00) |
|  | Women | | | | |
|  | 82 | 82 | NR | NR | 0.85 (0.76-0.95) |
|  | **COMPARE TO AWGS2** | | | | |
| Lin et al. (2021) | Men | | | | |
|  | 94.83 | 56.41 | 94.83 | 0.88 | 0.86 (, 0.78-0.94) |
|  | Women | | | | |
|  | 82.05 | 85.71 | 82.05 | 0.89 | 0.85 (0.77-0.94) |
| Ding et al. (2023) | Men | | | | |
|  | 93.2 | 59.1 | 79.07 | 83.8 | 0.83 (0.75 to 0.90) |
|  | Women | | | | |
|  | 93.3 | 64.7 | 52.83 | 95.6 | 0.84 (0.77 to 0.93) |
| Chen et al. (2020) | Men | | | | |
|  | Original cut-off point | | | | |
|  | 64.94 | 85.46 | 64.94 | 92 | NR |
|  | New cut-off point | | | | |
|  | 70.65 | 81.35 | 70.65 | 92 | 0.81 (0.75 to 0.86) |
|  | Women | | | | |
|  | Original cut-off point | | | | |
|  | 46.91 | 93.22 | 46.91 | 90 | NR |
|  |  |  | New cut-off point |  |  |
|  | 75.31 | 79.9 | 75.31 | 94 | 0.84 (0.80 to 0.89) |
| Zhu et al. (2022) | Men | | | | |
|  | 85 | 77.2 | 85 | 0.88 | 0.82 (0.74-0.91) |
|  | Women | | | | |
|  | 96.3 | 88 | 96.3 | 0.92 | 0.83 (0.73-0.92) |
|  | **COMPARE <2 CONSENSUS** | | | | |
| Locquet et al. (2017) | EWGSOP criteria | | | | |
|  | 89.70 | 80.9 (76.5–85.3) | 89.70 | 96.3 (94.2–98.4) | 0.85 (0.80–0.90) |
|  | EWGSOP2 criteria | | | | |
|  | 84.3 (80.2–88.4) | 77.7 (73.0–82.4) | 84.3 | 97.7 (96.0–99.4) | 84.3 (80.2–88.4) |
|  | IWGS criteria | | | | |
|  | 86.8 (83.0–90.6) | 74.3 (69.4–79.2) | 86.8 | 97.7 (96.0–99.4) | 86.8 (83.0–90.6) |
|  | Society of Sarcopenia, Cachexia, and Wasting Disorders criteria | | | | |
|  | 100.0 (100–100) | 74.1 (69.2–79.0) | 100.0 | 100.0 (100–100) | 100.0 (100–100) |
|  | AWGS criteria | | | | |
|  | 100.0 (100–100) | 74.9 (70.0–79.8) | 100.0 | 99.1 (98.0–100) | 100.0 (100–100) |

**Supplemental file 01.** The accuracy of the Ishii score chart in predicting sarcopenia. Notes: NR: Not reported. EWGSOP: European Working Group on Sarcopenia in Older People; IWGS: International Working Group on Sarcopenia; AWGS: Asian Working Group for Sarcopenia; FNIH: Foundation for the National Institutes of Health; PPV: positive predictive value; NPV: negative predictive value; AUC, area under the curve.
