# Supplementary material for: Use of the Ishii Test for screening sarcopenia in older adults: a systematic review with meta-analysis of diagnostic test accuracy (DTA) studies
Source: BMC Geriatr. 2024 Jul 17;24:609. doi: 10.1186/s12877-024-05155-2 (PMC11253494; doi:10.1186/s12877-024-05155-2)
Supplement: Supplementary file 2 — Supplementary Material 2. [file 12877_2024_5155_MOESM2_ESM.docx]

Figure S1. Comparison of the accuracy between the Ishii test and different diagnostic methods for men.


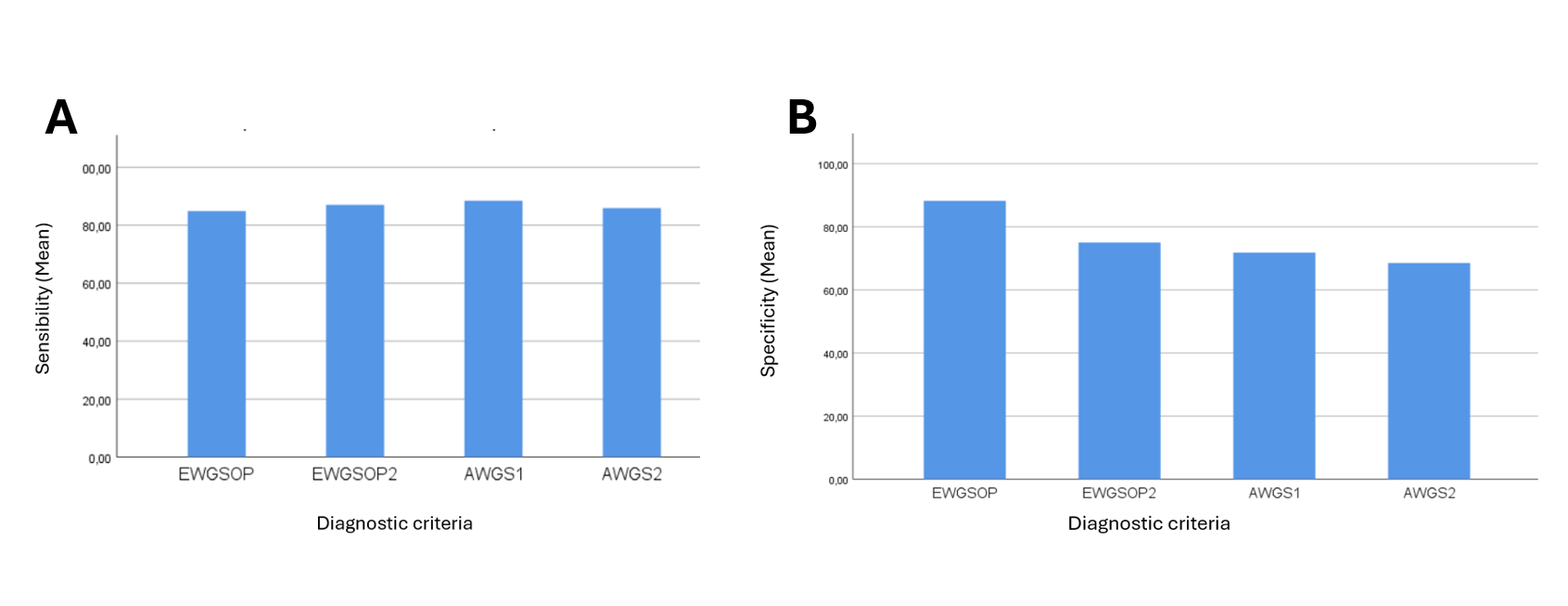


Note: A = Comparative results of sensibility; B = Comparative results of specificity
